# In Silico and In Vivo Analysis of IL37 in Multiple Sclerosis Reveals Its Probable Homeostatic Role on the Clinical Activity, Disability, and Treatment with Fingolimod

**DOI:** 10.3390/molecules25010020

**Published:** 2019-12-19

**Authors:** Eugenio Cavalli, Emanuela Mazzon, Maria Sofia Basile, Santa Mammana, Manuela Pennisi, Paolo Fagone, Reni Kalfin, Vanja Martinovic, Jovana Ivanovic, Marko Andabaka, Sarlota Mesaros, Tatjana Pekmezovic, Jelena Drulovic, Ferdinando Nicoletti, Maria Cristina Petralia

**Affiliations:** 1 IRCCS Centro Neurolesi “Bonino-Pulejo”, Via Provinciale Palermo, Contrada Casazza, 98124 Messina, Italy; eugenio.cavalli@irccsme.it (E.C.); emanuela.mazzon@irccsme.it (E.M.); santa.mammana@irccsme.it (S.M.); m.cristinapetralia@gmail.com (M.C.P.); 2 Department of Biomedical and Biotechnological Sciences, University of Catania, Via S. Sofia 89, 95123 Catania, Italy; sofiabasile@hotmail.it (M.S.B.); manuela.pennisi@unict.it (M.P.); paolofagone@yahoo.it (P.F.); 3 Institute of Neurobiology, Bulgarian Academy of Sciences, Acad. G. Bonchev Str., Block 23 1113 Sofia, Bulgaria; reni_kalfin@abv.bg; 4 Clinic of Neurology, Clinical Center of Serbia, Dr Subotica 6, 11000 Belgrade, Serbia; vanja_martinovic@yahoo.com (V.M.); jo_ivanovic@yahoo.com (J.I.); marko_med@yahoo.com (M.A.); sharlotam@gmail.com (S.M.); pekmezovic@sezampro.rs (T.P.); drulovicjelena@gmail.com (J.D.)

**Keywords:** cytokines, interleukin 37, multiple sclerosis, fingolimod

## Abstract

We evaluated the in silico expression and circulating levels of interleukin (IL)37 in patients with different forms of multiple sclerosis (MS) and also upon treatment with different disease-modifying drugs. The combined interpretation of the resulting data strengthens and extends the current emerging concept that endogenous IL37 plays an important role in determining onset and progression of MS. The in silico analysis revealed that production of IL37 from cluster of differentiation (CD)4+ T cells from MS patients was reduced in vitro as compared to healthy controls. The analysis of the datasets also demonstrated that “higher” levels of IL37 production from PBMC entailed significant protection from MS relapses. In addition, the in vivo part of the study showed that IL37 was selectively augmented in the sera of MS patients during a relapse and that treatment with the high potency disease-modifying drug fingolimod significantly increased the frequency of patients with circulating blood levels of IL37 (6/9, 66%) as compared to patients receiving no treatment (*n* = 48) or platform therapy (*n* = 59) who had levels of IL37 below the limit of the sensitivity of the assay. This finding therefore anticipates that fingolimod may at least partially exert its beneficial effects in MS by upregulating the production of IL37.

## 1. Introduction

Multiple sclerosis (MS) is a chronic autoimmune disease of the central nervous system (CNS), characterized by demyelination and neurodegeneration [1], which represents the most frequent neuroinflammatory disease in young adults, with a mean age of diagnosis of about 30 years and a worldwide prevalence rate of 30.1 cases per 100,000 population in 2016 [2].

We and others have shown that proinflammatory cytokines such as macrophage migration inhibitory factor (MIF), interleukin (IL)12, and IL18 can be implicated in progressive forms of the disease [3,4,5,6,7,8] and that an altered balance between proinflammatory Th1 and Th17 cytokines and anti-inflammatory Th2 and Th3 cytokines may represent important pathogenetic events contributing to the onset and clinical course of relapsing remitting forms of the disease, as well as its therapeutic response. Indeed, endogenous anti-inflammatory mediators such as IL1-receptor antagonist (IL1-Ra, encoded by the gene IL1RN) [9], soluble IL1-receptor [10], transforming growth factor (TGF)-β [11], and IL4 can be augmented by disease-modifying drugs such as interferon (IFN)-β or glatiramer acetate [12]. Both glatiramer acetate and IFN-β reduce IL17A [13]. We have also shown that corticosteroids that are used during relapses of the disease increase the circulating levels of soluble IL1 type II receptor and IL1-Ra [10].

Hence, a tightly regulated endogenous network composed of proinflammatory and anti-inflammatory cytokines and other cellular and soluble mediators may control the onset and the progression of the disease and may also be implicated in its therapeutic response [14].

IL37 is a cytokine belonging to the IL1 family that was originally discovered by in silico research [15]. IL37 can be found in the cytoplasm and nucleus [16] and is detectable in many human cells, including monocytes; natural killer (NK) cells; stimulated B cells; and tissues such as thymus, bone marrow, lymph nodes, colon, lung, and uterus [17]. The human IL37 gene is located on chromosome 2 and undergoes alternative splicing. There are five splice variants from IL37a to IL37e. IL37b, the longest sequence in the five isoforms of IL37, contains 218 amino acids and represents the cytokine expressed at maturity [18]. To exert its biological function, IL37 binds to a receptor complex composed of single Ig IL-1-related receptor (SIGIRR), also known as IL1-R8 or Toll interleukin-1 receptor 8 (TIR8) and IL18-Rα congregated on the surface of peripheral blood mononuclear cells (PBMCs) upon pro-inflammatory stimulation [19]. IL37 may also interact with low affinity and without influencing the biological function of IL18 with the α receptor subunit of IL18 receptor (IL18Rα or IL18R1). However, the inhibition of IL18 proinflammatory activity results from the binding of IL37 with the IL18 binding protein (IL18BP) and with IL18-Rβ, which results in an inactive complex that reduces the production of IFN-γ levels [16]. Once bound to the receptor, IL37 is translocated to the nucleus, where it interacts with Smad3 before activating specific gene transcription [20].

In addition, it was recently demonstrated that human IL37 has a caspase-1 cleavage site and that nuclear translocation of IL37 is inhibited by caspase-1 inhibition [21].

Several preclinical studies support the concept that IL37 may potently downregulate immune-inflammmatory responses. Among others, these include the anti-inflammatory function of IL37 in human coronary artery endothelial cells [22], as well as the augmented production of proinflammatory cytokines in human peripheral blood mononuclear cells secondary to IL37 knockdown [20]. Moreover, treatment with recombinant human IL37 suppresses lipopolysaccharide (LPS)-induced cytokines IL1-β, IL6, and tumor necrosis factor (TNF)-α; inhibits p38 mitogen-activated protein kinase (MAPK) activation in M1-differentiated human blood macrophages; and reduces innate inflammation markers in mice subjected to endotoxemia [23]. In rodent models of autoimmune diseases, the intra-articular injection of an adenovirus vector expressing IL37 into the knee joints of mice with type II collagen-induced arthritis (CIA) markedly down-regulated the clinical and histological signs of arthritis [24].

Other studies have shown that IL37 exerts beneficial anti-inflammatory effects in animal models of colitis and myocardial ischemia-reperfusion injury and concanavalin A (ConA)-induced immunoinflammatory hepatitis [25,26]. In the clinical setting, it has concordantly been shown that circulating and/or PBMC levels of IL37 are increased in several autoimmune diseases including systemic lupus erythematosus (SLE), rheumatoid arthritis (RA), inflammatory bowel disease, ankylosing spondylitis, psoriasis, and Graves’ disease [25]. These increased levels often occur during exacerbations of the diseases and correlate with biomarkers of disease activity, suggesting that the expression of IL37 corresponds to the disease activity of RA, ankylosing spondylitis (AS), Graves’ disease (GD), and SLE [25,26,27,28]. Of additional interest was the observation that in vitro-added IL37 decreased production of proinflammatory cytokines from PBMCs of AS, GD, and SLE patients, but not of healthy controls [25]. These augmented levels of IL37 in inactive phases of autoimmune diseases have been interpreted as a compensatory attempt that is physiologically activated by the body to counteract excessive ongoing immunoinflammatory responses. Nonetheless, even though it is inconsistent with the potent anti-inflammatory action of IL37 in experimental studies, the hypothesis can neither be ruled out that the augmented levels of IL37 may play an unexpected pathogenetic role in these autoimmune diseases by activating unknown inflammatory pathways that have so far not been recognized to be modulated by IL37. Cytokines exerting primarily anti-inflammatory effects have subsequently been shown to be also capable of upregulating immunoinflammatory responses, as has been shown with endogenous IL4 in models of autoimmune hepatitis [29] and exogenously administered IL10 in models of orchitis [30]. Vice versa, anti-inflammatory effects of prototypical Th1 cytokines such as IFN-γ and also TNF-α have been reported in models of type 1 diabetes [31,32].

In spite of these data of IL37 in autoimmune diseases, only a limited number of studies have evaluated the possible role of this cytokine in MS [33,34,35,36]. Farrokhi et al. showed that IL37 serum levels were higher both in patients suffering from relapsing-remitting MS (RR-MS) and those with neuromyelitis optica (NMO) as compared to healthy controls. In addition, they noticed a positive correlation between the serum levels of IL37 and Expanded Disability Status Scale (EDSS) of patients [33]. These findings were subsequently confirmed in another independent study that demonstrated that serum levels of IL37 were higher in patients with MS and correlated with disease activity as well as with the levels of IL33 and soluble vascular endothelial growth factor (VEGF) receptor 2 [34].

Taken together, these papers seem to indicate that endogenous IL37 is produced in response to ongoing immunoinflammatory events during the course of MS with the possible, and yet unsuccessful, attempt to downregulate the progression of the disease. Along this line of research, recent data have shown that the beneficial effects of hypoxia-preconditioned human periodontal ligament cell secretome in myelin oligodendrocyte glycoprotein peptide (MOG)-induced experimental autoimmune encephalomyelitis (EAE) were associated with marked expression of IL37 [35].

These initial data propelled us to further study the involvement of IL37 in MS. To do so, we evaluated in silico expression and circulating levels of IL37 in patients with different forms of the disease and also upon treatment with different disease modifying drugs [37]. The combined interpretation of the resulting data strengthens and extends the current emerging concept that endogenous IL37 plays an important role in determining onset and progression of MS. In addition, the present demonstration that fingolimod, but not other disease-modifying therapies (DMTs), augmented the levels of IL37 in a significant manner indicates that this drug may at least partially exert its beneficial effects in MS by upregulating the production of IL37.

## 2. Results

### 2.1. IL37 Expression in Peripheral Cluster of Differentiation (CD4)+ T Cells from MS Patients and Healthy People

**Figure 1:**
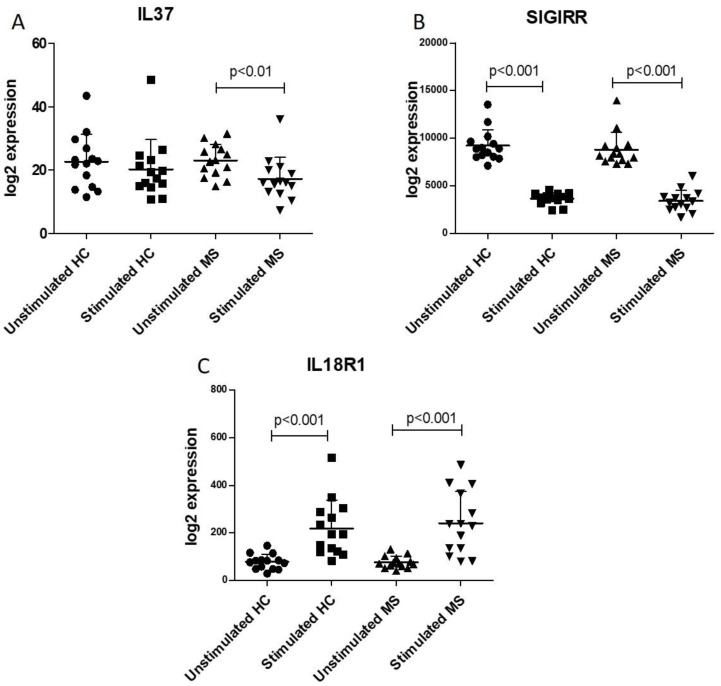
Analysis of circulating cluster of differentiation (CD)4+ T cells from multiple sclerosis (MS) patients and healthy controls (HC). Transcriptomic profiles of resting and activated CD4+ T cells in patients with multiple sclerosis and healthy donors was obtained from the GSE78244 dataset. Expression levels of interleukin (IL)-37 (**A**), SIGIRR (**B**) and IL18R1 (**C**) were evaluated from unstimulated cells and upon 24 h anti-CD3/CD28 antibody stimulation.

We found no difference in the expression levels of IL37 and of the receptors, SIGIRR and IL18R1, when comparing CD4+ T cells from MS and healthy controls, both in basal and stimulated conditions (Figure 1).

However, unlike the CD4+ T cells from healthy controls that secreted comparable amounts of IL37 both upon resting conditions and upon stimulation with anti-CD3 + antiCD28, this stimulation of the CD4+ T cells from MS patients determined a significant (*p* < 0.01) reduction in IL37 secretion (Figure 1A). Superimposable reduction in SIGIRR levels was observed in T helper cells, both from MS patients and healthy controls (*p* < 0.01) (Figure 1B). On the other hand, IL18R levels significantly (*p* < 0.001) increased following T cell activation in both CD4+ T cells isolated from both MS patients and healthy donors (Figure 1C).

### 2.2. IL37 Expression during Stable and Relapsing Disease

**Figure 2:**
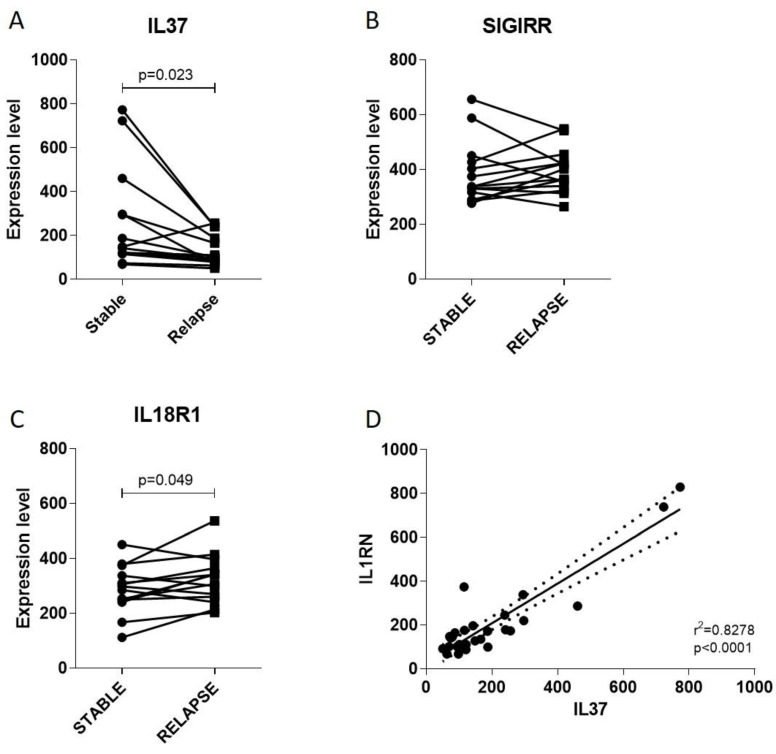
Evaluation of IL37 (**A**) and its receptors SIGIRR (**B**), IL18R1 (**C**) and IL1RN (**D**) during MS relapse. Gene expression profiles of peripheral blood mononuclear cells (PBMCs) of MS patients in stable and relapsing disease was obtained from the publicly available microarray dataset, GSE19224.

In order to evaluate whether a modulation in IL37 levels could be observed during clinical relapse of MS, we interrogated the GSE19224 dataset. As shown in Figure 2A, a significant reduction in IL37 expression was observed in PBMCs from MS patients undergoing exacerbation of the disease (*p* = 0.023). No modulation was observed for SIGIRR (Figure 2B), whereas a moderate but significant increase (*p* = 0.049) in IL18R1 expression was found (Figure 2C). A significant correlation between IL37 and the anti-inflammatory factor, IL1RN, was also observed (Figure 2D).

### 2.3. IL37 Expression in Lymphocytes from Monozygotic Twin Pairs Discordant for MS

**Figure 3:**
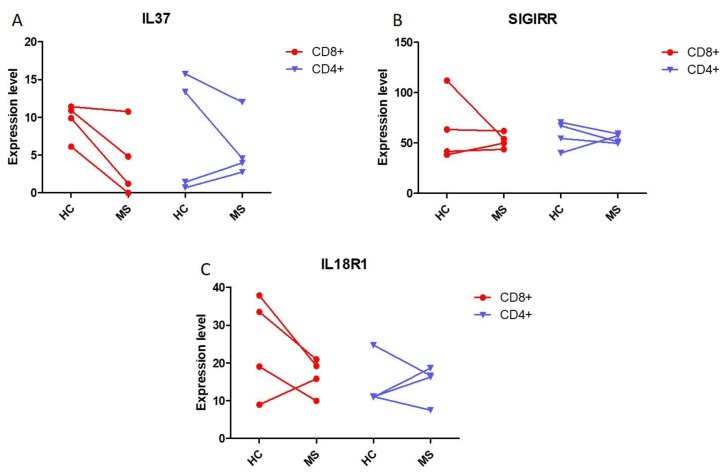
Evaluation of IL37 (**A**) and its receptors SIGIRR (**B**) and IL18R1 (**C**) in monozygotic twin pairs discordant for MS. To determine the expression levels of the genes of interest in peripheral CD4+ and C8+ T cells from monozygotic twins discordant for MS, the GSE16461 dataset was interrogated.

The expression levels of IL37, SIGIRR, and IL18R1 were evaluated in monozygotic twin pairs discordant for MS. As shown in Figure 3, a trend to reduced levels for the three analyzed genes was observed in CD4+ and CD8+ T cells isolated from the MS-affected individuals; however, no statistical significance was reached, probably because of the very limited number of subjects studied (Figure 3).

### 2.4. Prediction of Relapses by Transcription Levels of IL37 and Its Receptors

**Figure 4:**
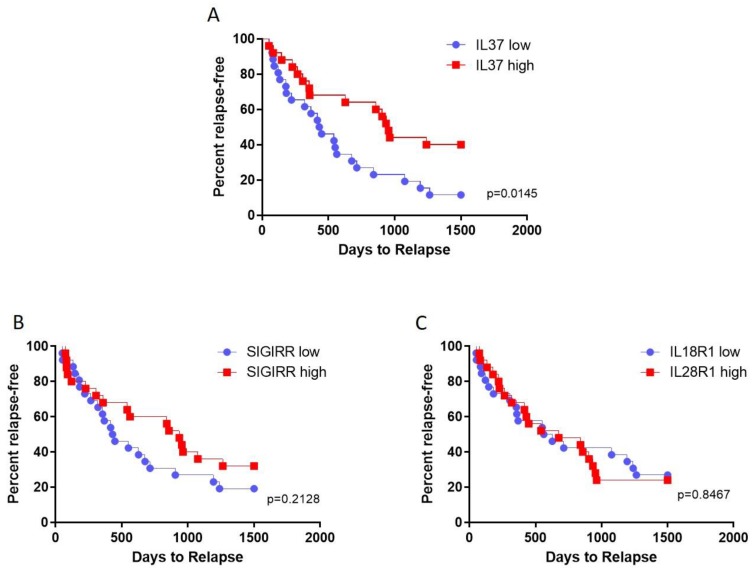
Prediction of MS relapses by transcription levels of IL37 and its receptors in PBMCs. Patient population was divided into two groups on the basis of the expression level of each of the genes of interest (referred to as high and low expression) and survival curves generated for an observational period of 1500 days. IL37 (**A**), SIGIRR (**B**), and IL18R1 (**C**) were considered in the analysis. Data were retrieved from the freely accessible GSE15245 microarray dataset.

We next evaluated whether the different transcriptional levels of IL37 and its receptors in PBMCs from MS patients could promote or protect MS patients from acute relapses. The patient population was divided into two groups on the basis of the expression level of each of the genes of interest (referred to as high and low expression) and survival curves generated for an observational period of 1500 days. As shown in Figure 4, higher levels of IL37 entailed a significant protection to the exacerbation of the disease (*p* = 0.0145) (Figure 4). On the other hand, no influence on relapse occurrence was observed for SIGIRR and IL18R1 (Figure 4).

### 2.5. Analysis of IL37 in Sera from MS Patients

IL37 was detected in the sera from 11 out of the 127 recruited MS patients. In particular, IL37 could be detected in 1 clinically isolated syndrome (CIS) patient (concentration: 616.953 pg/mL) in 8 out of the 95 RR-MS patients (one sample was lost due to technical reasons) and in 2 out of 8 secondary progressive MS (SP-MS) patients. None of the patients with primary progressive MS (PP-MS) had detectable IL37 in sera (Table 1). No statistical significance was reached for the differences in the frequency of dosable IL37 among the groups of patients.

Non-parametric correlation analyses between serum IL37 levels and clinical parameters in MS patients are presented in Table 2.

Higher IL37 levels were associated with younger age (*p* = 0.047) and lower Multiple Sclerosis Severity Score (MSSS; *p* = 0.039). Correlations with other demographic and clinical parameters did not reach the statistical significance. All of the eight patients with dosable levels of IL37 were under treatment with the DMTs. In particular, six of them were treated with fingolimod, one with ocrelizumab, and one with glatiramer acetate. The highest levels of IL37 (3564, 1144, and 963 pg/mL) were detected in RR-MS patients treated with fingolimod. We observed a significantly augmented proportion of samples with dosable circulating IL37 in MS patients treated with high potency DMTs compared to those untreated (36.36% vs. 0%; *p* = 0.0007, by chi square test with Yates’ correction) and compared to patients treated with platform therapy (36.36% vs. 0%; *p* = 0.0001, by chi square test with Yates’ correction). Moreover, a significantly higher proportion of samples with dosable circulating IL37 was detected comparing MS patients treated with fingolimod with patients treated with platform therapy (66.66% vs. 0%; *p* = 0.0001) and with patients untreated (66.66% vs. 0%; *p* = 0.0001). When comparing the serum IL37 levels between patients with RR-MS and SP-MS, we observed a trend towards a higher median level in RR-MS in comparison with SP-MS patients (*p* = 0.059). We next observed a significantly augmented proportion of samples with dosable circulating IL37 in patients suffering from an acute exacerbation of MS as compared to those with stable disease (34.6% vs. 8.4%; *p* = 0.0127, by chi square test with Yates’ correction). Corticosteroid treatment was associated with a trend to both increased numbers of patients with detectable levels of IL37 (42.3% vs. 34.6%), as well as with increased concentrations (Table 3).

**Figure 5:**
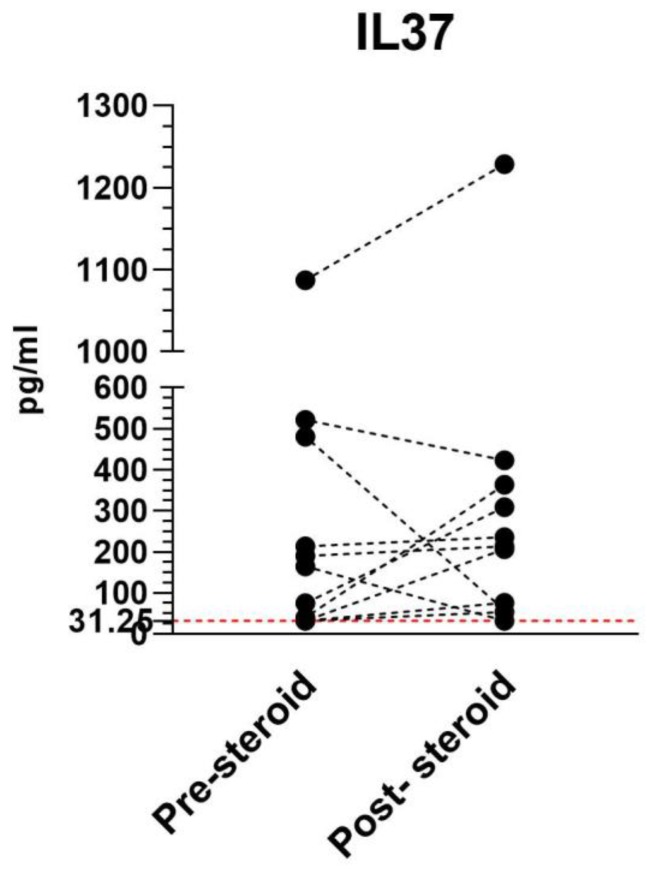
Serum IL37 levels in 11 RR-MS patients before and after short-term, high-dose steroids.

In particular, upon corticosteroid treatment, we observed an increase in serum IL37 concentration in eight patients, and a decrease in three patients (Figure 5). However, none of the trends were statistically significant.

## 3. Discussion

In the present study, we first analyzed the expression levels of IL37, SIGIRR, and IL18R1 in circulating immune cells from MS patients. In particular, we performed a DNA microarray analysis that represents a useful in silico tool for the better understanding of pathogenic pathways and the possible prediction of novel diagnostic therapeutic strategies, as it has been shown in a variety of clinical settings, such as autoimmune and immunoinflammatory diseases [38,39,40,41,42,43,44] and cancer [45,46,47,48,49,50,51], leading to the identification of novel therapeutic targets [52,53,54,55].

Following activation, CD4+ T cells from MS patients showed lower levels of expression of IL37 than helper T cells from healthy controls. Opposite results were observed for the IL37 receptors. Indeed, in stimulated CD4+ T cells, either in healthy control or in MS patients, we observed a downregulation of SIGIRR and an upregulation of IL18R1. Conversely, in unstimulated CD4+ T cells, both in healthy control or in SM patients, SIGIRR was upregulated and IL18R1 was downregulated.

Moreover, we found a reduction of IL37 levels in PBMCs from MS patients undergoing a relapse compared to stable patients. We also investigated the level expression of IL18R1 and SIGIRR, which showed only a moderate or no modulation between patients with stable or relapsing disease.

Of particular interest, in the in silico analysis had the observation that higher levels of IL37 levels in PBMC entailed a significant protection to the exacerbation of the disease.

It is noteworthy that our in silico data show how IL37 and IL1Ra are directly correlated. In PBMCs from MS patients undergoing a relapse, the mutual levels increase of IL1Ra and IL37 was observed. This finding adds IL37 to the IL1 family of endogenous anti-inflammatory networks composed from IL1Ra-soluble IL1 receptor type II that we and others have previously studied in MS [56]. The above data suggest that the elevated levels of endogenous IL1Ra and IL37 may indicate an attempt to promote anti-inflammatory responses, possibly inhibiting immuno-inflammatory events induced by IL1.

In addition to the data elaborated from the in silico study, we also generated new data in an in vivo study by analyzing IL37 serum level in MS patients with different phenotype conditions and different therapeutic responses. We studied IL37 levels in patients with PP-, SP-, and RR-MS in stable disease or during relapses. It has been generally accepted that inflammatory processes within the CNS in MS are more exaggerated in the younger population [57], and therefore our findings that higher IL37 levels were associated with younger age (*p* = 0.047) are in line with this notion. Additionally, we demonstrated that higher IL37 levels were associated with lower MSSS (*p* = 0.039), thus supporting the previously mentioned hypothesis that IL37 produced in response to ongoing immunoinflammatory events in MS might downregulate the progression of the disease. Therefore, the activation of IL37 and IL1Ra could be crucial in limiting the harmful effects of IL1 during the recovery phase in MS.

Furthermore, our data demonstrate that the serum levels of IL37 were significantly augmented during relapses of the disease, as well as in patients with stable disease upon treatment with highly effective DMT. In particular, the greatest percentage of MS patients with stable disease exhibiting dosable levels of IL37 was found in the MS patients treated with fingolimod, with six out of nine having detectable levels of IL37 in their circulation. It seems therefore possible that one of the immunopharmacological mode of action by which fingolimod ameliorates RR-MS may rely on the induced production of IL37. In vitro and in vivo studies are necessary to more precisely dismantle the mode of action of fingolimod on IL37 production. Previous studies on the immunopharmacological mode of action of this drug in RR-MS have shown that it increases in a non-specific fashion both pro- and anti-inflammatory cytokine-producing T helper subsets (IFN-γ, TNF-α, IL4, and IL10-producing CD4+ T cells) [58]. We also observed an increasing trend of IL37 level expression after steroid therapy. These data could indicate that IL37 and IL1Ra are closely related, demonstrated by the fact that glucocorticoids and transforming growth factor (TGF)-β are among the agents that reduce the production of proinflammatory cytokines of the IL1 family [59].

The combined comparison of in silico analysis and in vivo data appear in conflict with regard to the finding that a significant reduction in IL37 expression was observed in PBMCs from MS patients undergoing exacerbation of the disease in the in silico analysis whereas augmented levels of IL37 were observed in the circulation of MS patients suffering an exacerbation. These apparently conflicting results may be explained by the fact that in the in silico analysis only the production of IL37 from PBMC was evaluated, whereas measurement of the circulating levels of IL37 also takes into account the production of IL37 from other non-immune sources including uterus and gastrointestinal cells for example [17]. Although our study is the first to demonstrate increased levels of IL37 during relapses of MS, two previous studies found augmented blood levels of IL37 in Iranian patients suffering from RR-MS and NMO. It is very difficult to compare the studies and dismantle reasons for the possible differences. In particular, in the two studies in the Iranian population, it was not specified whether the patients were sampled during stable phases of MS or during exacerbations. Information on the treatment of the patients was not provided, yet this information is clearly essential as DMT modifies the course of the disease and the counter-inflammatory responses that could evoke IL37 production.

Our results thus indicate that IL-1Ra activation and expression of IL37 could be one of the defense mechanisms leading to remissions in MS and lower disease severity as measured by MSSS. Further studies are warranted to understand whether the dysregulated balance between pro-inflammatory and anti-inflammatory cytokines, their receptors, and endogenous inhibitors might play a role in designing novel diagnostic tools and therapeutic approaches for MS. Indeed, our present work represents only an observational study and cross sectional analysis for the treatment arm on the effects of DMTs on IL-37 levels, and no direct or causal relation between the observed level of IL37 and the clinical parameters can be presently inferred. Nonetheless, our study adds new important pieces of new information to the potential role of IL37 in MS and its eventual contribution to mediate the action of fingolimod on the course of the disease.

## 4. Materials and Methods

### 4.1. In Silico Analysis: Microarray Selection

The whole-genome transcriptomic profile of resting and activated CD4+ T cells from MS patients and healthy controls was obtained from the GSE78244 dataset [60], downloaded from the public databank Gene Expression Omnibus (GEO; https://www.ncbi.nlm.nih.gov/gds). The expression levels of the genes of interest were evaluated in unstimulated cells and upon 24 h incubation with anti-CD3/CD28 antibodies. The microarray dataset included data from 14 RR (relapse remitting)-MS patients and 14 healthy donors. All the patients were women and none of them had received immunomodulatory/immunosuppressive drugs for at least 2 months, except for one who underwent intravenous immunoglobulin 15 days before sampling. No relapse was experienced by any of the patients in the prior 3 months [60]. The Agilent-039494 SurePrint G3 Human GE v2 8 × 60K Microarray platform was used and data preprocessing was performed by the Feature Extraction software version 10.7.3.1 (Agilent Technologies, Santa Clara, California, United States), using default parameters (protocol GE1-107_Sep09) [60]. Gene expression profiles of PBMCs of MS patients in stable and relapsing disease were obtained from the publicly available microarray dataset, GSE19224 [61]. The dataset included 14 patients with RR-MS [61]. The relapse samples were obtained within the first week of clinical exacerbation and before initiation of corticosteroid treatment. The relapse was defined as the occurrence of new symptoms or the worsening of existing symptoms—including hemiparesis, paraparesis, sensory loss in various distributions, unilateral ataxia, and myelopathy—with a duration of at least 48 h, in the absence of other illness. The stable samples were obtained at least 1 month before or 3 months after a clinical relapse. The female/male ratio was 9:5. Six patients were on IFN-β treatment at both sampling times, two patients were on no disease-modifying treatment, four patients were receiving glatiramer acetate at both sampling times, and two patients were untreated at the time of relapse and on glatiramer when the stable samples were obtained [61]. The dataset was generated using Illumina HumanRef-8 v3.0 expression beadchip [61]. To determine the expression levels of the genes of interest in peripheral CD4+ and C8+ T cells from monozygotic twins discordant for MS, we interrogated the GSE16461 dataset, originally published by [62]. The mean age was 37.5 ± 2.9 and the female/male ratio was 3:1. The male twin comprised an SP patient. All of the other patients suffered from RR-MS. All patients were treatment-naïve [62]. The dataset was generated using the Affymetrix Human Genome U133 Plus 2.0 Array. Linear Model for Microarray (LIMMA) analysis was used for the evaluation of statistical significance.

In order to evaluate the relationship between expression levels of the genes of interest and the time to relapse, we interrogated the GSE15245 dataset, which included the transcriptional profile of PMBCs from 51 drug-naïve clinically definite MS patients [63]. Patients had a mean age of 38.5 ± 1.4, and a mean EDSS score of 2.4 ± 0.2 [63]. The dataset was generated using the Affymetrix Human Genome U133A 2.0 Array and data were preprocessed using the robust multi-chip average (RMA) background correction algorithm. The sample population was sorted on the basis of the expression levels of IL37, SIGIRR, and IL18R1. Log-rank test was applied to evaluate differences in the percentage of patients developing acute relapses in a 1500 day time-frame (Table 4).

### 4.2. Patients

A total of 129 consecutive MS patients (40 male and 89 female) from the Department for Central Nervous System (CNS) Immune-Mediated Disorders, Clinic of Neurology, Clinical Center of Serbia, Belgrade, were enrolled in the study. The diagnosis was established according to the revised McDonald criteria 2017 [64]. Mean age at examination was 41.1 ± 10.1 years (mean ± SD) with disease duration of 11.1 ± 7.5 years. The demographic and clinical characteristics of MS patients are presented in Table 5.

The study comprised patients with various MS phenotypes established according to the criteria of Lublin (2004) [65]. The majority of patients (85%) suffered from RR-MS in clinical remission.

The level of neurological disability was assessed by the Expanded Disability Status Scale (EDSS) [66]. In addition, we also evaluated the Multiple Sclerosis Severity Score (MSSS) in each patient in order to rate disease severity using disability as measured by EDSS and disease duration [67]. MSSS corrects EDSS for duration by using an arithmetically simple method to compare an individual’s disability with the distribution of scores in cases having equivalent disease duration. A reference table for comparisons was created by applying the previously mentioned method to a database of 9892 European MS patients. The MSSS is a useful method for identifying factors that influence disease progression using single assessment data.

RR-MS patients were treated with approved DMTs. The first group was treated with moderate efficacy DMTs (“platform therapy”) that included IFN-β-1b, IFN-β-1a subcutaneous (sc) 3 times per week, IFN-β-1a intramuscular (im), glatiramer acetate, and teriflunomide. The second group was treated with high efficacy DMTs and included fingolimod, natalizumab, ocrelizumab, alemtuzumab, and mitoxantrone. No corticosteroid treatment was used for these patients during the 6 months before their inclusion in the study.

Out of these 96 patients presenting with stable RR-MS at initial blood sampling, 26 suffered from an acute exacerbation during the period of completion of recruitment into the study. Relapse was defined as the occurrence of a new neurological disturbance with a duration of at least 24 h [68]. These patients had been treated 3–10 days after the onset of the new neurological disturbances with short intravenous (i.v.) course of high-dose steroids for 2 h in the early morning (methylprednisolone, 1 g/day for 5 days). Serum samples were obtained from all patients just before the initiation of the treatment (pre-treatment) and at the day after the end of the treatment (day 6). The informed consent was obtained from each patient prior to inclusion in the study. The study was approved by the Ethical Committee of the Clinical Centre of Serbia, Belgrade (N° 890/6, dated 21.12.2018).

### 4.3. ELISA

IL37 concentration in sera from MS patients was quantified with an ELISA kit (Cusabio, Houston, TX, USA). The detection range of the kit was 31.25–2000 pg/mL. All assay procedures were carried out according to the manufacturer’s instructions. The absorbance values of standards and samples were obtained at 450 nm (reference wavelength 540 nm) using a VICTOR Nivo Multimode Microplate Reader. For evaluation of statistical analysis, the samples with levels of IL37 below the limit of sensitivity of the assays were assigned this limit as a theoretical value.

### 4.4. Statistical Analysis

For the analysis of the correlation between serum IL37 levels and clinical parameters in MS patients, the non-parametric Spearman test was used. To determine the significance of the different proportions of patients with dosable IL37, a chi square test with Yates’ correction was used. A two-tailed Wilcoxon matched-pairs signed rank test was used for the evaluation of differences in IL37 before and after corticosteroid treatment. Two-tailed *p*-values less than 0.05 were considered significant. Data were analyzed using the Statistical Package for the Social Sciences (SPSS) software (Advanced Statistics, version 17.0, Chicago, IL, USA) and GraphPad Prism software (San Diego, CA, USA).

## Figures and Tables

**Table 1:** IL37 levels in sera from MS patients.

Variable		IL37 in Sera		
MS Phenotypes		Proportion Detected *	Proportion Not Detected *	Level (pg/mL) Median, IQR
CIS		1/10 (10.0)	9/10 (90.0)	616.953
RR-MS	Stable	8/95 (8.4)	87/95 (91.6)	613.349, 954.357
	Exacerbation	11/26 (42.3)	15/26 (57.7)	
SP-MS		2/8 (25.0)	6/8 (75.0)	60.451, 77.046
PP-MS		0/14 (0.0)	14/14 (100.0)	
Total		22/153 (14.4)	131/153 (85.6)	

* Number (%); MS: multiple sclerosis; CIS: clinically isolated syndrome; RR-MS: relapsing-remitting MS; SP-MS: secondary progressive MS; PP-MS: primary progressive MS; IQR: interquartile range.

**Table 2:** Correlation between sera IL37 levels and clinical parameters in 11 MS patients in whom sera IL37 was detected.

Variable	Correlations	
	Correlation Coefficient (ρ)	*P*
Gender	0.000	1.000
Age	−0.609	**0.047**
EDSS	−0.222	0.597
Disease duration	−0.429	0.188
MSSS	−0.733	**0.039**
Treatment history	0.107	0.755

EDSS: Expanded Disability Status Scale; MSSS: Multiple Sclerosis Severity Score. Significantly value are in bold.

**Table 3:** IL37 levels in sera from RR-MS patients in relapse before and after short-term, high-dose steroids.

Variable	IL37 in Sera	
	Proportion Detected *	Level (pg/mL) Median, IQR
Before steroids	9/26 (34.6)	119.331, 418.069
After steroids	11/26 (42.3)	220.793, 389.109

* Number (%); IQR: interquartile range.

**Table 4:** Clinical characteristics of the patients from the microarray datasets.

	GSE78244	GSE19224	GSE16461	GSE15245
Number of MS patients	14	14	4 twins	51
Number of healthy controls	14	-	4 co-twins	-
Gender	All females	9 females 5 males	6 females 2 males	35 females 16 males
Age of MS patients (years)	39.9 ± 13	-	39.2 ± 4.5	38.5 ± 1.4
Age of healthy controls (years)	40.4 ± 8.9	-	39.2 ± 4.5	-
MS phenotypes	RR-MS	RR-MS stable/relapse	3 RR-MS1 SP	-
EDSS score	-	-	-	2.4 ± 0.2
Treatment history	None	6 interferon 2 untreated 4 glatiramer acetate 2 untreated (relapse)/glatiramer (stable)	None	None

**Table 5:** Characteristics of multiple sclerosis patients.

Variable	
Number	129
Gender *	
Male	40 (31.0)
Female	89 (69.0)
Age (years)	
Mean ± SD	41.1 ± 10.1
MS phenotypes *	
CIS	10 (7.8)
RR-MS	96 (74.3)
SP-MS	9 (7.0)
PP-MS	14 (10.9)
Disease duration (years)	
Mean ± SD	11.1 ± 7.5
EDSS score	
Median (range)	2.5 (0.0–8.5)
MSSS	
Mean ± SD	3.7 ± 2.3
Treatment history	
No treatment	48 (37.2)
Platform therapy	59 (45.7)
High potency	22 (17.1)

* Number (%).
